# A nomogram as a predictive tool for lymph node metastasis in papillary thyroid carcinoma

**DOI:** 10.3389/fendo.2026.1850799

**Published:** 2026-06-16

**Authors:** Yimeng Liu, Tianxiang Liu, Yi Long, Xin Wang, Mei Tao, Kejia Xu, Xing Wan, Jimin Zhao, Guangwei Xu, Jie Gao, Yongjun Piao, Xuan Qin, Xiangqian Zheng

**Affiliations:** 1 Tianjin Medical University Cancer Institute & Hospital, National Clinical Research Center for Cancer, Tianjin’s Clinical Research Center for Cancer, Tianjin Key Laboratory of Basic and Translational Medicine on Head & Neck Cancer, Tianjin, China; 2 School of Medicine, Nankai University, Tianjin, China

**Keywords:** lymph node metastasis, nomogram, PHQ-9, prediction, thyroid cancer

## Abstract

Lymph node metastasis is the most prevalent form of spread in thyroid cancer, with surgical intervention and prophylactic cervical lymph node dissection being the standard treatments. However, there remains a lack of effective methods for predicting lymph node metastasis in clinical practice. The study enrolled patients who were admitted to the Department of Thyroid and Neck Oncology at Tianjin Medical University Cancer Hospital between October 2021 and March 2022, as well as from February to April 2024. Variables collected included basic patient information, laboratory tests, and pathological data. Additionally, we focused on investigating the impact of emotional problems on lymph node metastasis. Subsequently, variable selection was performed using Least absolute shrinkage and selection operator (LASSO) regression and multivariate logistic regression analyses. A nomogram was then constructed for predicting lymph node metastasis, with model performance assessed using calibration curves, decision curves and other methods. In this study, a total of 484 cases were ultimately included, and the variables were screened using LASSO regression. Subsequently, nine variables were utilized to construct a nomogram. For model training purposes, 85% of the data was randomly selected as the training set and the prediction efficiency was verified using ROC curve analysis. The AUC index for both the training set (0.8045) and verification set (0.8146) were obtained along with calibration curve and decision curve plots. This study developed a nomogram to predict lymph node metastasis in papillary thyroid cancer. This model demonstrates exceptional discrimination and calibration, providing invaluable assistance for clinical decision-making processes.

## 1 Introduction

Thyroid cancer is a prevalent endocrine malignancy that arises from the thyroid gland, an organ which is located in the neck and can produce hormones to regulate metabolism (1).

Thyroid cancer incidence rates have increased, it now ranks as the fifth most frequently diagnosed cancer in women globally (2, 3). The male-to-female ratio of thyroid cancer incidence is approximately 1:3.3, with the age of onset ranging from 14 to 80 years old, and the peak age of incidence being between 30 and 59 years old. Among Chinese women, thyroid cancer exhibits the fastest-growing incidence rate of all cancers (4). Based on pathological characteristics, thyroid cancer is broadly categorized into differentiated and undifferentiated types. The differentiated category encompasses papillary thyroid carcinoma (PTC) and follicular thyroid carcinoma, with PTC being the predominant form, representing approximately 60% of differentiated cases and 84% of all thyroid cancers (5, 6).

Papillary thyroid cancer occurs more frequently in middle-aged and older women than in men (5). Lymph node metastasis (LNM) is the predominant metastatic pathway in papillary thyroid cancer, with the cervical region being the primary site of involvement (7). While most patients with papillary thyroid cancer have an excellent prognosis after undergoing surgery, those with LNM face a significantly poorer outcome (8). Consequently, the primary therapeutic approach involves surgical intervention coupled with prophylactic neck lymph node dissection.

Currently, there is a notable absence of clear clinical standards regarding the decision-making process for patients considering complete cervical lymph node dissection (9). Inadequate treatment may result in tumor recurrence and metastasis, while excessive treatment can adversely affect the patient’s quality of life (10). Furthermore, performing an extensive cervical lymph node dissection elevates the risk of complications, notably hypoparathyroidism and recurrent laryngeal nerve injury. Therefore, it is imperative to establish a robust approach for the accurate assessment of LNM, thereby mitigating the risks of both missed diagnoses and overtreatment (11).

Artificial intelligence has emerged as a powerful tool for detecting predictive signals and early risk factors for numerous diseases. With their ability to capture complex nonlinear relationships, machine learning algorithms are increasingly being used to develop predictive models that can inform clinical decision-making (12, 13), including recent multicenter nomograms (14).

Currently, several studies have attempted to predict LNM in PTC using clinicopathological variables alone or combined with imaging features (15–17). However, to our knowledge, no predictive model has integrated routine hematological biomarkers—specifically platelet count (PLT) and activated partial thromboplastin time (APTT)—with clinicopathological parameters for preoperative LNM risk stratification. Emerging evidence suggests that platelets and coagulation factors may influence tumor microenvironment and metastatic potential through mechanisms involving immune evasion and angiogenesis (18–20). Therefore, we hypothesized that incorporating PLT and APTT into a comprehensive nomogram could enhance predictive accuracy and provide a readily accessible preoperative assessment tool.

Beyond traditional biomedical factors, accumulating evidence indicates that psychological status, particularly emotional distress (ED), may influence cancer progression and metastasis through neuroendocrine and immune pathways (21, 22). The Patient Health Questionnaire-9 (PHQ-9), a validated instrument for assessing depression severity, has been associated with treatment response and survival outcomes in various malignancies (23). However, its predictive value for lymph node metastasis in PTC remains unexplored. We therefore incorporated preoperative PHQ-9 assessment to investigate whether emotional distress constitutes an independent dimension of LNM risk in PTC patients.

Despite these advances, several critical gaps remain in the current literature. First, existing nomograms for PTC LNM prediction rely primarily on postoperative pathological or preoperative imaging data, limiting their applicability in resource-limited settings where advanced imaging is unavailable. Second, routine hematological parameters—despite their low cost and universal availability—have not been systematically evaluated as predictors of LNM in PTC. Third, the potential contribution of preoperative psychological status to LNM risk stratification has been entirely overlooked. To address these gaps, the present study developed and validated a novel nomogram integrating clinicopathological variables, hematological biomarkers (PLT and APTT), and psychological assessment (PHQ-9) for preoperative prediction of LNM in PTC. This represents, to our knowledge, the first attempt to incorporate multidimensional preoperative data—including psychological factors—into a clinically applicable prediction model for PTC.

This study initially determined the determinants of lymph node involvement in papillary thyroid carcinoma. The predictive model established and validated in this study represents the first endeavor specifically aimed at quantifying the risk of lymph node metastasis for this cancer type. We recommend that clinicians utilize this nomogram to facilitate guidance and assistance in subsequent treatment decisions.

## 2 Materials and methods

### 2.1 Patients

A cohort of 612 patients, hospitalized in the Department of Thyroid and Neck Oncology (Tianjin Medical University Cancer Hospital) across three distinct periods (Oct–Nov 2021, Feb–Mar 2022, Feb–Apr 2024), constituted the study population.

Inclusion criteria were as follows:

1. Laboratory-related examinations and thyroid function tests were required to be performed prior to surgery;
2. Papillary thyroid cancer must be confirmed through postoperative histopathological examination;
3. Patients must have undergone radical surgical treatment for thyroid cancer within our department.

Exclusion criteria were as follows:

1. Incomplete laboratory or clinical pathology data;
2. Benign diseases other than thyroid cancer, which encompass benign nodules, benign tumors, nodular goiter, retrosternal goiter, adenomatous goiter, benign tumors of the submandibular gland or parotid gland, Warthin tumor of the parotid gland, peripheral cervical nerve tumors, autonomic neurogenic tumors, thyroglossal duct cysts, hyperthyroidism, lipomas, regional lymphadenopathy, Hashimoto’s thyroiditis, atypical thyroid hyperplasia, cervical chylous fistula, parathyroid adenomas or benign parathyroid tumors, and cervical thymomas of type AB;
3. Other types of thyroid cancer or tumor recurrence, excluding papillary thyroid cancer, which include follicular adenoma of the thyroid, medullary thyroid cancer, follicular or metastatic follicular thyroid carcinoma, borderline thyroid tumors, oncocytic thyroid tumors, recurrent thyroid cancer, and lymph node metastases from thyroid cancer;
4. Other malignant tumors or tumor recurrence, such as lymphoma, thymic junctional tumors, secondary malignant tumors of cervical or supraclavicular lymph nodes, and postoperative recurrence of parotid acinar carcinoma.
5. Application of the predefined inclusion and exclusion criteria yielded a final cohort of 484 cases for the follow-up investigation.

### 2.2 Variables

The variables collected in this study can be categorized into three main parts:

1. Basic information, including patient demographics such as age, gender, surgical history, and histories of smoking and alcohol use, etc.
2. Laboratory tests, comprising thyroid function tests, blood biochemistry, complete blood counts, and coagulation function tests, etc.
3. The postoperative pathological data, including tumor characteristics and the definitive pathological status of lymph node metastasis (LNM), served as the basis for our model, with this status defined as the dichotomous outcome variable.

This study further explored the role of emotional distress (ED) in papillary thyroid cancer.

Although ED has been associated with cancer recurrence and prognosis (21, 22), its relationship with lymph node metastasis is not well defined. Our objective was to assess whether ED contributes to nodal involvement in PTC. For this purpose, ED was measured with the Patient Health Questionnaire-9 (23).

### 2.3 Statistical analysis

Variables that most of the data were missing were excluded. Patients missing values in the remaining variables were also omitted from further analysis (24, 25). All analyses were conducted in R (v4.3.3) with two-sided tests and a significance level defined as p < 0.05. Continuous variables are expressed as mean ± standard deviation (SD), and categorical variables are summarized by counts and percentages. Depending on their distribution, continuous data were analyzed using either the t-test (for normally distributed data) or the Mann-Whitney U test (for non-normal data), while categorical data were assessed with the chi-squared test. A heatmap of Pearson correlation coefficients was first generated to visualize associations among variables. Following this, Least Absolute Shrinkage and Selection Operator (LASSO) regression was applied to refine the predictor set, with 10-fold cross-validation used to determine the optimal lambda value that minimized cross-validation error. LASSO calculated the regression coefficients to the model, and it involved a L1 penalty factor that reduced coefficients of some variables to zero to exclude insignificant variables. Variables with nonzero regression coefficients were considered relevant to Lymph node metastasis.

To ensure model stability and prevent overfitting, we calculated the events-per-variable (EPV) ratio for the final multivariate model. With 224 events (LNM-positive cases) in the training set and 9 independent predictors, the EPV ratio was 24.9, which exceeds the widely accepted minimum threshold of 10 events per variable for reliable logistic regression modeling.

Patients with selected features was divided into training set and test set, using an 85:15 ratio. A multivariate logistic regression analysis was applied to the training set to ascertain independent predictors of lymph node metastasis. Logistic regression was based on linear regression and sigmoid function was used to import nonlinear factor and clamp the value between 0 and 1 for binary classification. After modelling, a nomogram was plotted to transform regression formula to visual graph and display the importances of variables to the model. Evaluation of model discrimination was carried out on both training and test datasets via receiver operating characteristic (ROC) curves. Performance was summarized by the area under the curve (AUC), for which a 95% confidence interval was calculated using the Delong test. Calibration curve was utilized to compare predicted and actual probability and evaluate the calibration of model. The clinical application value of the model was investigated using decision curve analysis (DCA), which determines the net benefit over a spectrum of threshold probabilities. For categorical variables with sparse subgroups (T-stage IV, n=4; relationship=4, n=1; relationship=5, n=6), the large standard errors reflect limited sample sizes in these categories rather than model misspecification. Sensitivity analyses excluding these sparse subgroups demonstrated that the coefficients and significance of the remaining predictors remained stable (data not shown), indicating that these subgroups did not materially influence the overall model performance.

## 3 Results

### 3.1 General characteristics

**Figure 1:**
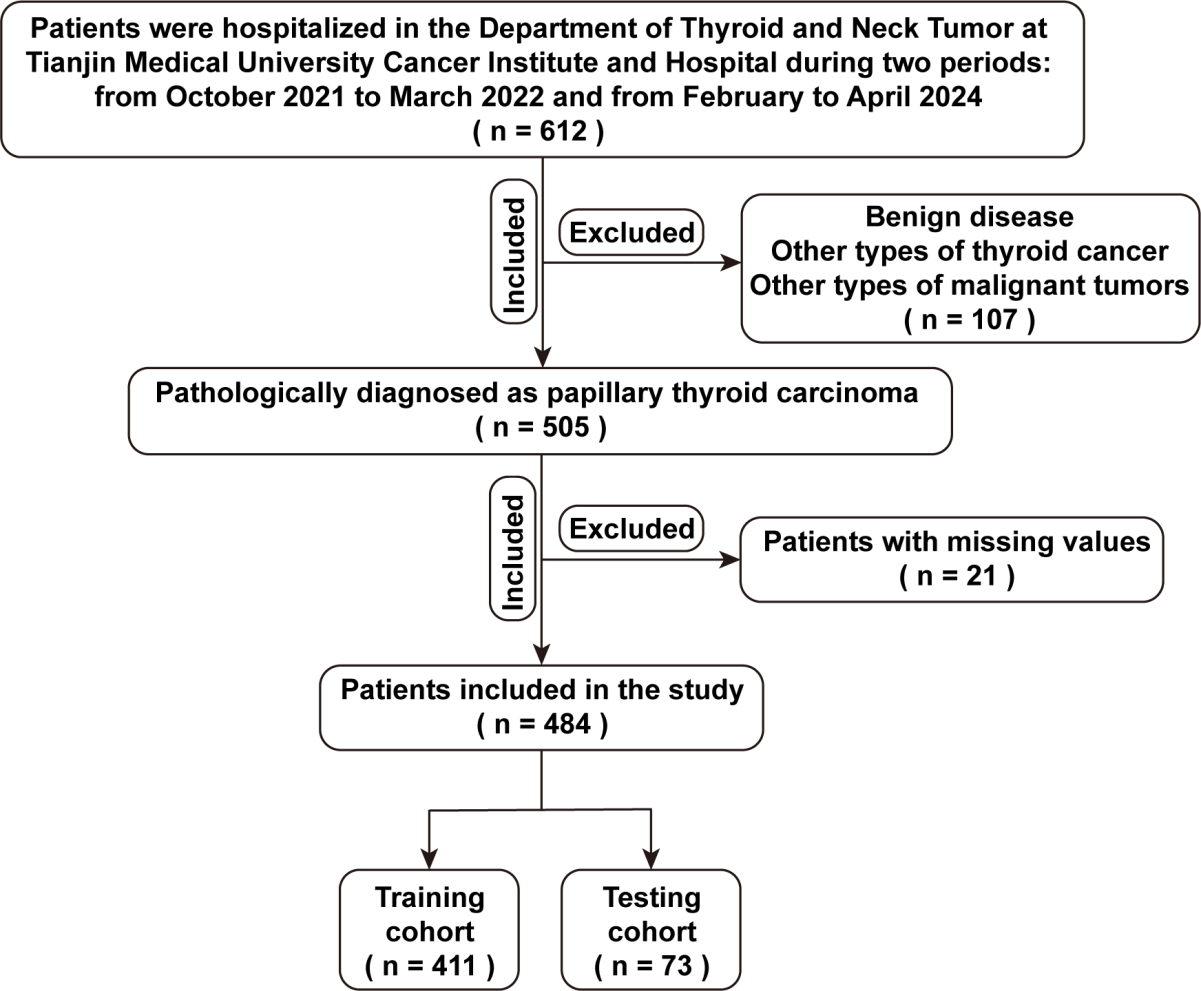
Flowchart of this study.

A schematic diagram of the study cohort selection is provided in **Figure 1**. The screening process initially identified 612 patients. After applying the predefined inclusion and exclusion criteria, 128 were excluded, yielding a final analytical cohort of 484 patients.

The final analysis cohort comprised 484 individuals diagnosed with papillary thyroid carcinoma.

The study cohort consisted of 484 patients, stratified by lymph node metastasis status (220 positive, 264 negative). **Table 1** and **Supplementary Table 1** summarizes the baseline characteristics according to this stratification. There are statistical differences in some variables.

**Table 1:** The baseline characteristics of patients categorized by the presence or absence of lymph node metastasis.

Variables	Total	Lymph-node-metastasis-no	Lymph-node-metastasis-yes	P value
	Total (n = 484)	0 (n = 220)	1 (n = 264)	
Baseline characteristics:				
Age, Median (Q1,Q3)	41 (34, 51)	46 (38, 53)	39 (32.75, 47)	< 0.001
Sex, n (%)				0.013
Female	360 (74)	176 (80)	184 (70)	
Male	124 (26)	44 (20)	80 (30)	
Pathological characteristics:				
T-stage, n (%)				< 0.001
T Ia	327 (68)	182 (83)	145 (55)	
T Ib	128 (26)	36 (16)	92 (35)	
T II	25 (5)	2 (1)	23 (9)	
T III	4 (1)	0 (0)	4 (2)	
Relationship, n (%)				< 0.001
within the gland	31 (6)	26 (12)	5 (2)	
close to fat	430 (89)	191 (87)	239 (91)	
close to striated muscle	16 (3)	3 (1)	13 (5)	
close to blood vessels	1 (0)	0 (0)	1 (0)	
infiltration of striated muscle	6 (1)	0 (0)	6 (2)	
Exocapsular invasion, n (%)				< 0.001
No	459 (95)	218 (99)	241 (91)	
Yes	25 (5)	2 (1)	23 (9)	
Maximum Tumor Diameter, Median (Q1,Q3)	0.8 (0.6, 1.2)	0.7 (0.5, 1)	1 (0.7, 1.5)	< 0.001
Tumor Counts, Median (Q1, Q3)	1 (1, 2)	1 (1, 2)	2 (1, 3)	< 0.001
Laboratory characteristics:				
PLT, Median (Q1, Q3)	235.5 (199, 275)	240 (204.75, 281)	230 (195.75, 264.25)	0.022
APTT, Median (Q1, Q3)	26.9 (25.2, 29.02)	26.65 (24.78, 28.92)	27.2 (25.8, 29.1)	0.01

In addition, we present the baseline characteristics of the 283 patients assessed using the PHQ-9 scale, as detailed in **Table 2**. PHQ-9 scores did not differ significantly between the LNM-positive and LNM-negative groups (mean 2.29 [SD 2.98] vs. 1.92 [SD 2.52], P = 0.272). Similarly, PHQ-9 score stratification showed no significant association with LNM status (P = 0.538, **Table 2**). Consequently, PHQ-9 was not retained in the final multivariate model. These null findings suggest that preoperative depression severity, as measured by PHQ-9, is not an independent predictor of LNM in PTC when adjusting for established clinicopathological and hematological variables.

**Table 2:** The baseline characteristics of the 283 patients assessed using the PHQ-9 scale.

	No	Yes	p
	n=130	n=153	
Lymph_node_metastasis = YES (%)	0 (0.0)	153 (100.0)	<0.001
PHQ-9 score_scale (%)			0.538
0<X ≤ 4	113 (86.9)	127 (83.0)	
4<X ≤ 9	15 (11.5)	21 (13.7)	
9<X ≤ 14	2 (1.5)	5 (3.3)	
14<X ≤ 20	0(0)	0(0)	
20<X	0(0)	0(0)	
PHQ-9 score [mean (SD)]	1.92 (2.52)	2.29 (2.98)	0.272

### 3.2 Identification of predictive factors for LNM

**Figure 2:**
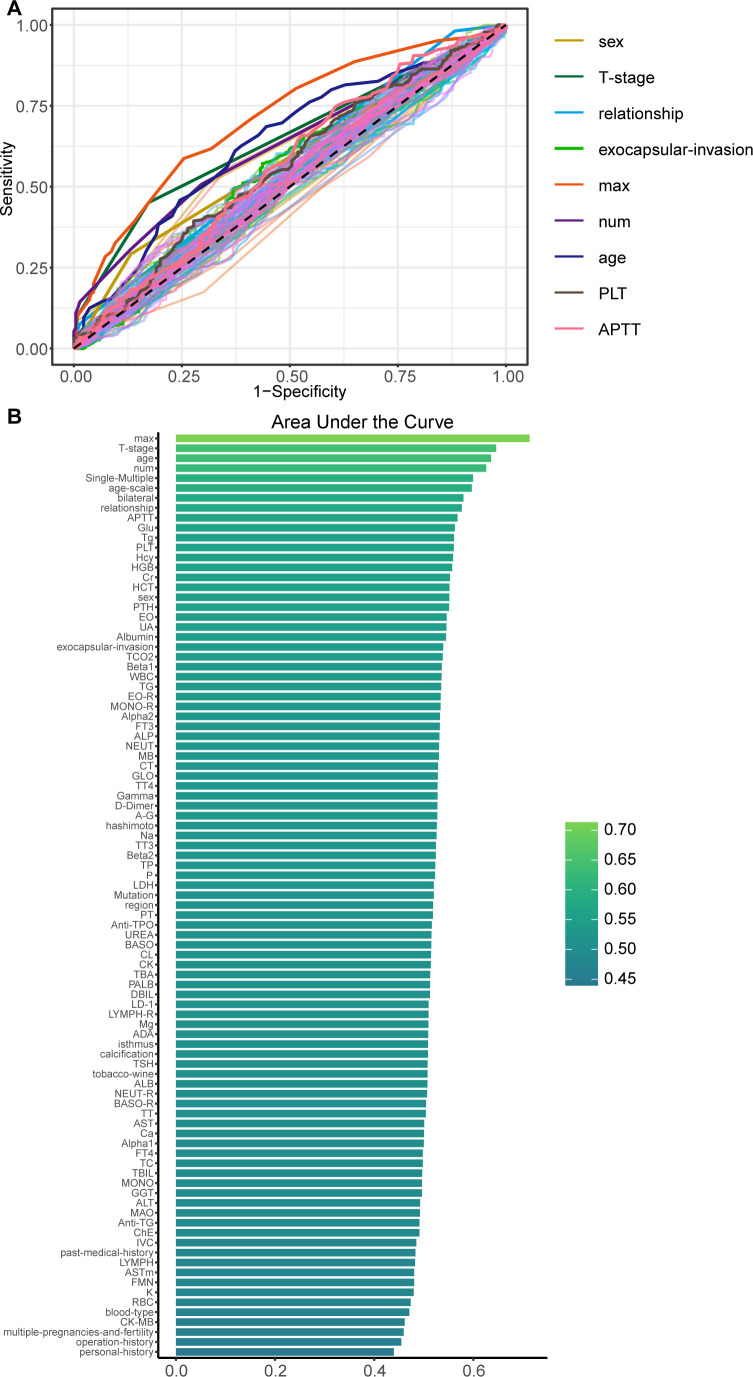
**(A)** ROC curves for all variables. Each curve represents the efficiency of each single variable in predicting the outcome. **(B)** The bar chart shows the area under the curve for each curve.

ROC curve analysis was performed to evaluate the predictive power of 94 variables for lymph node metastasis. Tumor size, T stage, and age demonstrated the highest AUC values (**Figures 2A, B**).

**Figure 3:**
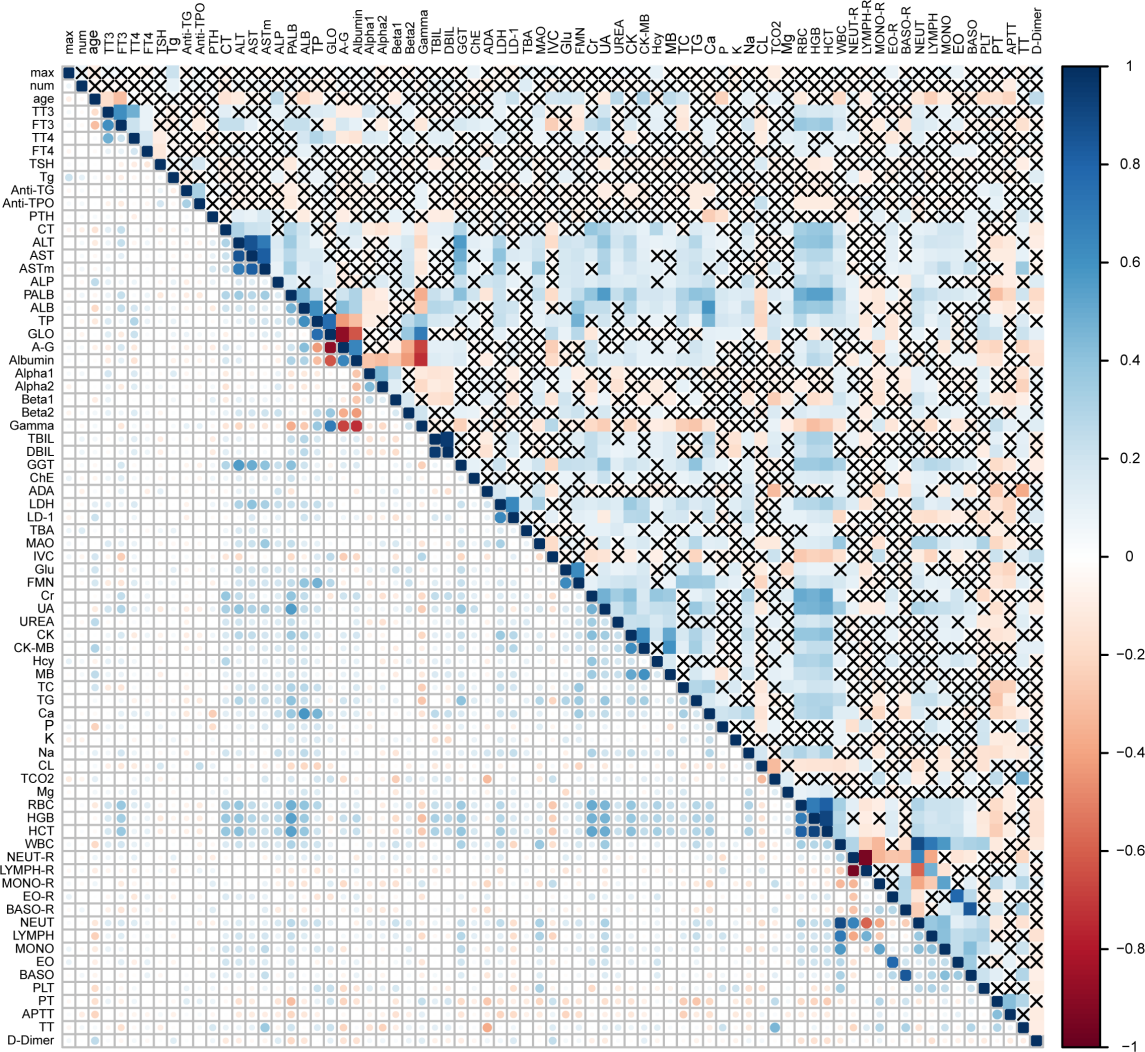
Heatmap of correlations between numerical variables.

To assess potential collinearity among the included variables, a correlation analysis was conducted on the numerical variables from the 94 variables in our dataset. This analysis resulted in the establishment of a correlation matrix and the generation of a correlation heat map (**Figure 3**). The findings indicate the presence of correlation and collinearity among these variables, suggesting that they cannot be directly included in logistic regression for predicting the presence or absence of LNM. Therefore, we will use lasso regression to filter the variables and select those most suitable for logistic regression.

**Figure 4:**
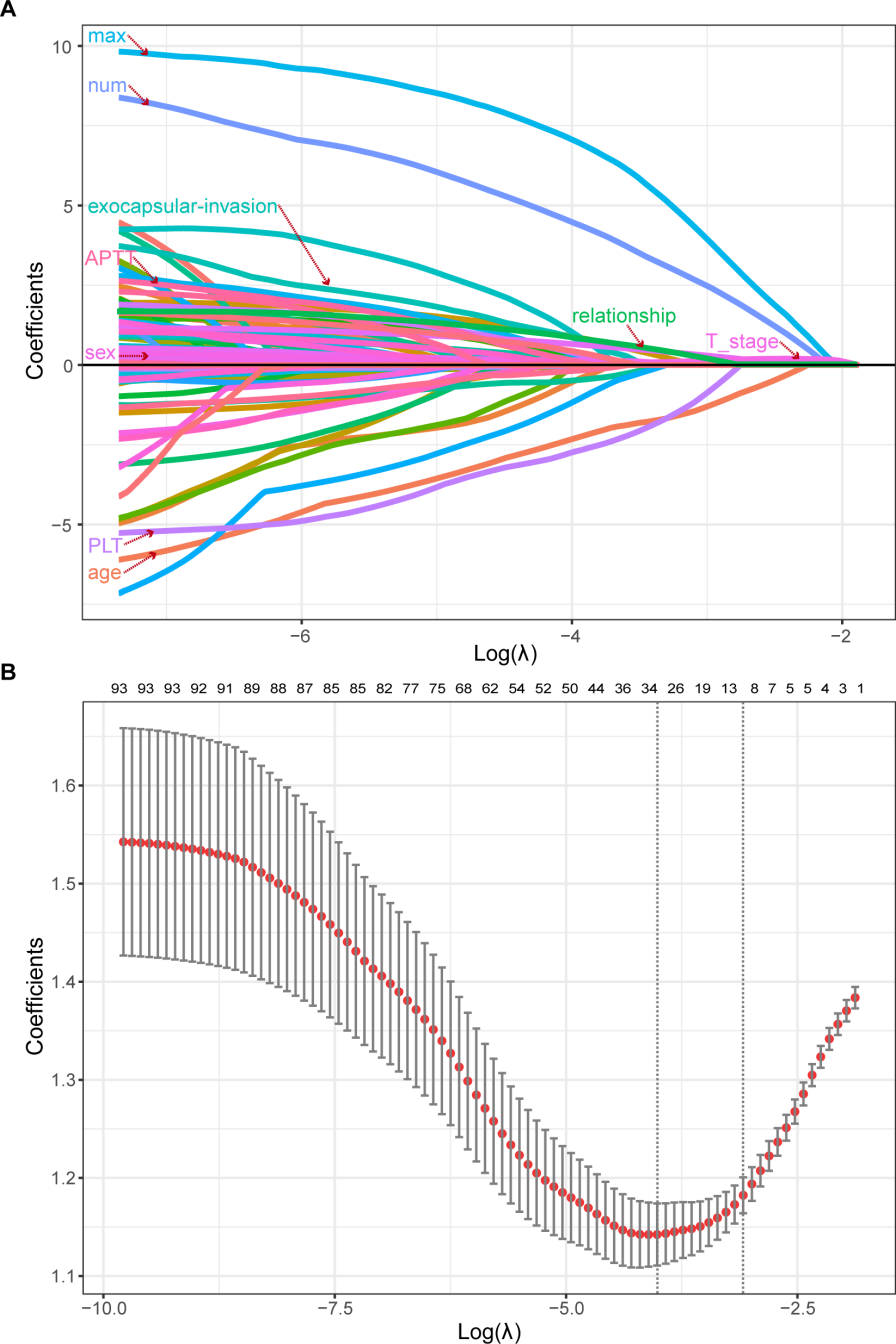
Screening of predictor variables using the LASSO regression analysis with 10-fold cross-validation. **(A)** LASSO coefficient profiles of the candidate variables. **(B)** Lambda (tuning parameter) selection of deviance in the LASSO regression based on the one standard error criterion (right dotted line) and the minimum criterion (left dotted line). The intersecting curves represent the number of features retained at that log(lambda) value, and ten predictors with nonzero coefficients were selected according to the one standard error criterion.

Given the high dimensionality and collinearity among variables, a two-step screening strategy was employed. LASSO regression initially identified ten candidate predictors (**Figures 4A, B**), which were subsequently entered into multivariate logistic regression. Nine independent predictors were retained in the final model (**Table 3**); single/multiple tumor occurrence was excluded due to non-significant regression coefficient.

**Table 3:** The multivariate analysis of lymph node metastasis in the training set.

	Coef	S.E.	Wald Z	Pr(>|Z|)
Intercept	-1.825	1.609	-1.130	0.257
sex=1	0.387	0.280	1.380	0.167
Single-Multiple=1	0.036	0.339	0.110	0.915
T-stage=2	-0.078	0.452	-0.170	0.863
T-stage=3	-0.205	1.194	-0.170	0.864
T-stage=4	-3.313	39.211	-0.080	0.933
relationship=2	1.304	0.598	2.180	0.029
relationship=3	2.277	0.934	2.440	0.015
relationship=4	9.166	60.987	0.150	0.881
relationship=5	5.988	27.901	0.210	0.830
Exocapsular invasion =1	2.466	1.152	2.140	0.032
max	1.390	0.494	2.820	0.005
num	0.344	0.125	2.750	0.006
age	-0.050	0.012	-4.230	<0.001
PLT	-0.006	0.002	-2.880	0.004
APTT	0.084	0.040	2.080	0.037

Large standard errors for T-stage = 4 (SE = 39.21), relationship = 4 (SE = 60.99), and relationship = 5 (SE = 27.90) reflect small sample sizes (n=4, 1, and 6, respectively). Sensitivity analyses excluding these subgroups did not materially alter the coefficients or significance of the remaining predictors.

### 3.3 Development of an individualized prediction model

**Figure 5:**
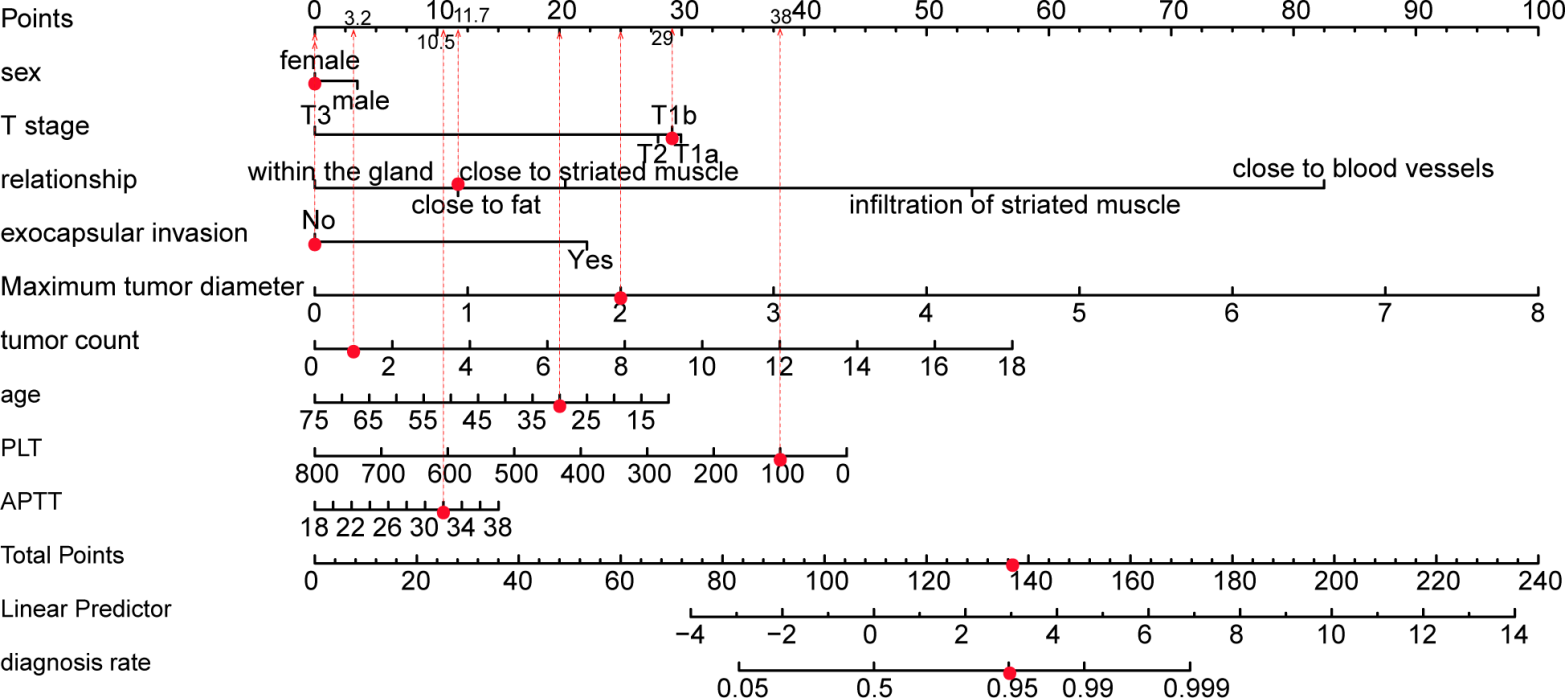
Nomogram for predicting the risk of lymph node metastasis. The red dots and dotted lines represent a patient example and the scores of the nomogram.

A nomogram incorporating the nine independent predictors was constructed (**Figure 5**). Maximum tumor diameter contributed the highest score, followed by tumor infiltration of striated muscle, tumor count, and platelet count.

For example, in the nomogram, a female patient, 30 years old, TNM stage Ib, tumor close to fat, Maximum Tumor Diameter is two centimeters, tumor counts 1, the value of PLT an APTT is 100 and 32. In this particular instance, the aggregate score is 137.4 points. Based on this aggregate score, the calculated probability of lymph node metastasis for this case is 0.95.

### 3.4 Predictive model validation

#### 3.4.1 Discrimination

**Figure 6:**
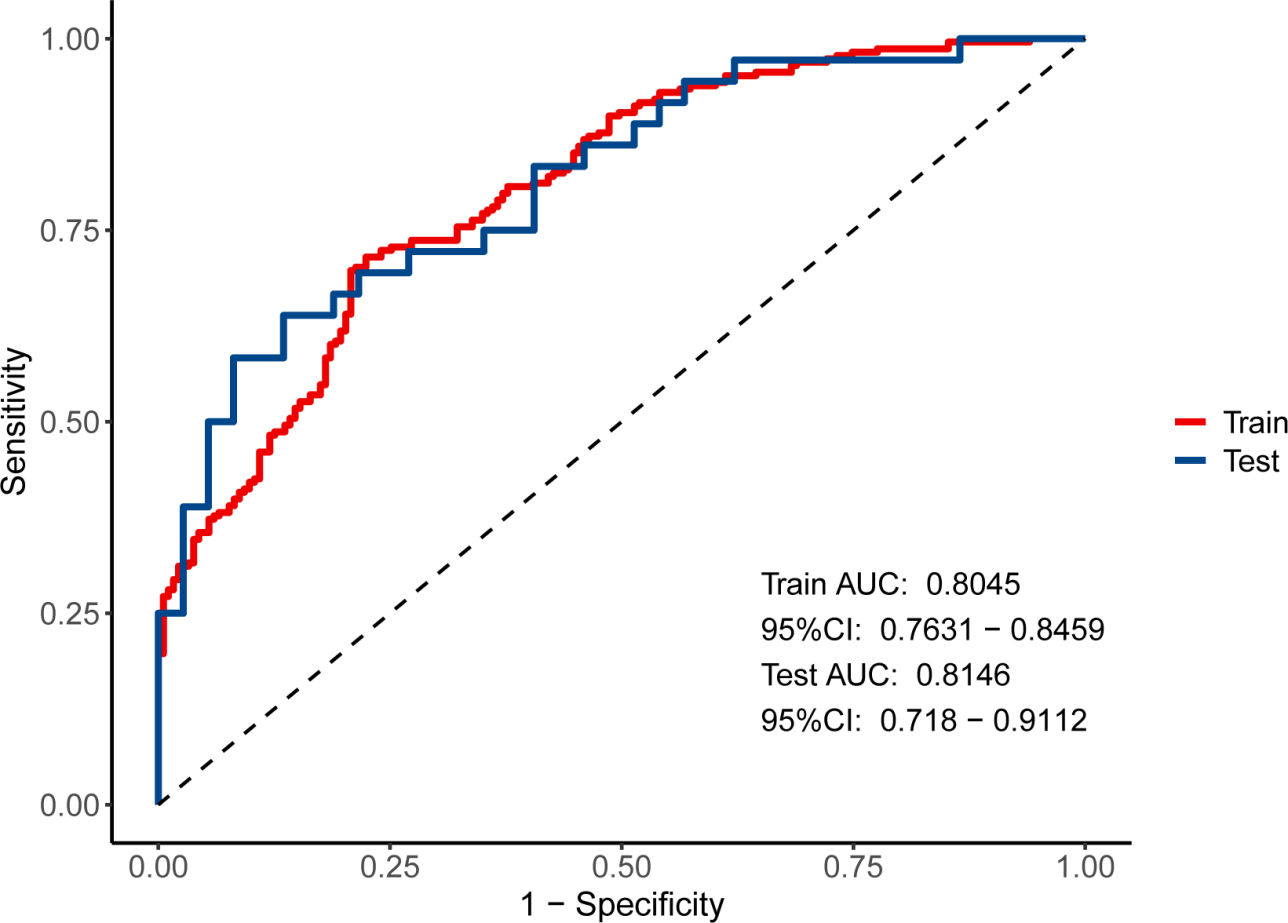
ROC curves showing the discriminatory ability of the model in the training set (red line, AUC = 0.8045) and validation set (blue line, AUC = 0.8146).

The nomogram achieved an AUC of 0.8045 (95% CI: 0.7631–0.8459) in the training set and 0.8146 (95% CI: 0.7180–0.9112) in the validation set (**Figure 6**), indicating good discriminatory performance.

#### 3.4.2 Calibration

**Figure 7:**
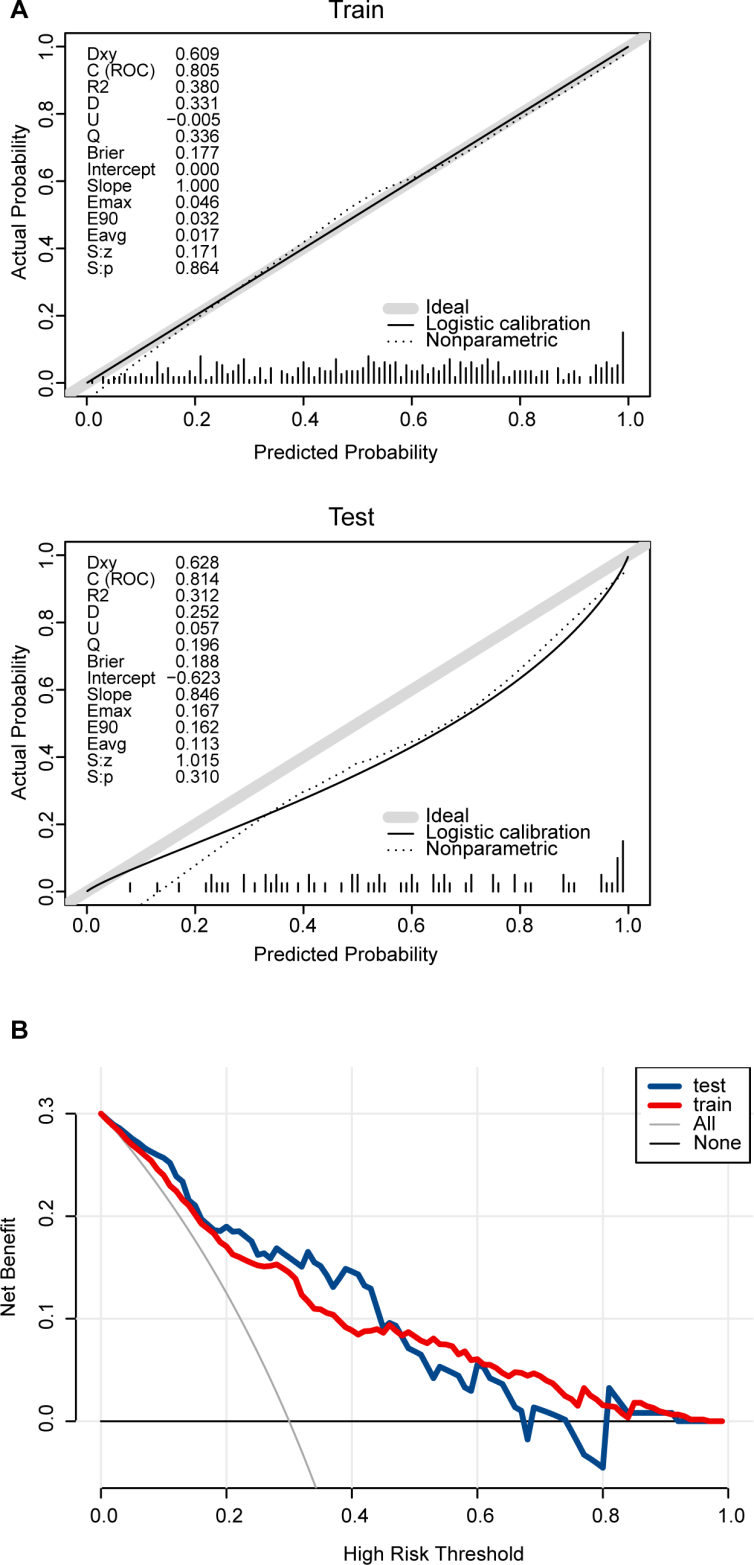
**(A)** Calibration curve of the predictive model showing the degree of consistency between the predicted probability and observed probability. The solid line represents a perfect prediction by an ideal model, and the dotted line shows the performance of the model. The shaded area represents the 95% confidence interval. **(B)** Decision curve analysis (DCA) for the nomogram. The black line represents the assumption of no lymph node metastasis, while the gray line assumes that all patients experienced lymph node metastasis. The analysis was conducted on both the training set (red line) and the validation set (blue line).

The calibration curves indicated good agreement between predicted and observed probabilities of lymph node metastasis across both training and validation cohorts (**Figure 7A**).

#### 3.4.3 Clinical use

The result of DCA indicated that the nomogram could yield significant net benefits for the patients with lymph node metastasis. The DCA results indicated that the nomogram provided significant net benefits for patients with lymph node metastasis. Net benefit was demonstrated over threshold probabilities of 1% to 100% in the training set. This clinical utility was maintained in the validation set for thresholds ranging from 1% to 68% (**Figure 7B**).

## 4 Discussion

With the continuous advancement of diagnostic technology, there has been an upward trend in the detection rate of thyroid cancer (26), accompanied by a concurrent rise in overdiagnosis (27). Despite the relatively favorable prognosis of thyroid cancer, early-stage cases often exhibit lymph node metastasis, which significantly affects patient outcomes. LNM is a crucial prognostic factor that influences the extent and approach to surgical intervention for papillary thyroid cancer, while also posing a substantial risk for high recurrence rates and diminished survival rates (28, 29). Consequently, due to the potential for LNM in patients, many individuals undergo total thyroidectomy and lymph node dissection (LND), resulting in widespread overtreatment (30).

Several studies have been proposed to stratify patients with papillary thyroid cancer by their clinically meaningful risk of lymph node metastasis. Factors such as tumor size, tumor location, tumor extension, microcalcifications, and Hashimoto ‘s disease are independent risk factors for lymph node metastasis (15, 16, 27, 31), as confirmed by population studies (32). Our research constructs a clinical prediction model in the form of a nomogram, which incorporates nine variables: gender, T stage, the relationship between the tumor and surrounding tissue, tumor exocapsular invasion, maximum tumor diameter, tumor number, age, platelet count (PLT), and activated partial thromboplastin time (APTT). The nomogram demonstrated good discriminatory ability and calibration, with DCA evaluations supporting its clinical utility. Our study included 484 patients, the majority of whom were female, comprising only 26% male participants, which is approximately one-third of the female population. Most of the enrolled patients were middle-aged, a demographic characteristic consistent with the prevalence of PTC, which is commonly observed in middle-aged women. These findings imply that the higher incidence of PTC in women does not logically entail a corresponding increase in their susceptibility to lymph node metastasis. In fact, a higher frequency of lymph node metastasis was observed among male patients relative to their female counterparts. Shen W et al. (33) corroborated this finding through a retrospective analysis, which established male gender as a significant clinical determinant for central lymph node metastasis (CLNM) in individuals with papillary thyroid carcinoma.

The present investigation age emerged as a significant predictor influencing LNM; younger patients were observed to demonstrate a significantly greater propensity for lymph node metastasis than females. Wang W et al. (34) corroborated these findings through a retrospective study involving a large cohort, indicating an inverse association between patient age and the likelihood of LNM. The size of a tumor is typically expressed by the maximum diameter of the tumor. This study demonstrates that patients with larger tumor diameters are more prone to lymph node metastasis, a finding consistent with numerous studies (17). In the TNM staging system, T I -T III are classified based on the tumor size; the larger the tumor, the later the T stage. In this study, patients with T1 and T2 had a higher risk of lymph node metastasis, while T3 was a protective factor against lymph node metastasis, which is inconsistent with the actual situation. This might be attributed to the relatively small sample size of T3, which could have led to prediction bias. The number of tumors is also a crucial risk factor influencing LNM. The investigation conducted by Konturek A et al. (35) discovered through multivariate analysis that multifocal PTC was an independent predictor of LNM. (OR = 3.99, 95% CI = 1.12 - 14.15, p = 0.033). Our findings align with existing literature (36), indicating that an increase in tumor count shows a positive association with lymph node metastasis development. However, our study only explored the impact of multifocal tumor presentation on LNM risk, neglecting the potential impact of tumor distribution, which warrants further investigation.

Tumor exocapsular invasion, defined as the infiltration of tumor cells beyond the thyroid capsule, was found to be closely related to lymph node metastasis. This study confirmed that tumor exocapsular invasion is an independent predictor. The association between the tumor and surrounding tissues is actually a specific division of the level of invasiveness. Among them, the proportion of tumors close to fat is the highest, accounting for 89%, while the proportions of tumors close to blood vessels, striated muscle, and the invasion of striated muscle are relatively low. However, the deeper the tumor invasion, the higher the risk of LNM. Previous literature has also demonstrated the same conclusion. Nie et al. compared the ultrasound images of LNM in PTC patients with computed tomography images. In the univariate analysis, their findings identified several tumor-related factors, including size, degree of invasiveness, and anatomical location, that demonstrated a significant relationship with LNM (P < 0.05) (37).

Reports on PLT (platelet count) and APTT (activated partial thromboplastin time) with cancer metastasis are currently relatively scarce. While they may not be directly linked to metastasis, PLT and APTT could influence tumor microenvironments, immune evasion, or coagulation pathways, indirectly affecting metastasis (18–20). Our study found that. lower PLT values and higher APTT values were linked to an elevated risk of LNM.

Finally, there is a potential predictor - patients’ emotional distress. While this variable was not selected for the final model, the observed difference in mean PHQ-9 scores between the groups is suggestive of an association between higher depression scores and lymph node metastasis.

The exploratory analysis of emotional distress yielded null findings, with PHQ-9 scores showing no significant association with LNM status. Several factors may explain this result. First, incomplete data collection (283/484, 58.5%) reduced statistical power to detect a modest effect. Second, the PHQ-9 assesses depression severity over the preceding two weeks, which may not capture chronic psychological states relevant to cancer biology. Third, lymph node metastasis in PTC is primarily driven by tumor biological characteristics (e.g., BRAF mutation, tumor size, extrathyroidal extension) rather than host psychological factors, potentially overshadowing any modest psychosomatic effect.

Despite these null findings, this analysis contributes to the limited literature on psychosomatic factors in thyroid cancer by demonstrating that PHQ-9-measured depression severity does not appear to be an independent predictor of LNM when established clinicopathological and hematological variables are considered. This observation underscores the need for prospective, multicenter studies employing comprehensive psychological batteries to definitively evaluate the role of emotional distress in PTC metastasis. Future studies should employ comprehensive psychological batteries (e.g., GAD-7 for anxiety, SF-36 for quality of life) and prospective designs to definitively evaluate the psychosomatic landscape in thyroid cancer.

This investigation is subject to several limitations. First, the data originate from a single clinical center, with no multi-center data collected for model construction. Second, all cases in this study are retrospective, highlighting the necessity for more prospective studies. The single-institution derivation of the dataset precluded external validation on independent cohorts, thus confining all accuracy assessments to internal verification methods. Future efforts should therefore focus on assembling multi-center datasets to enable rigorous external validation of the proposed model. Third, concerning the assessment of emotional distress, only 283 patients completed the PHQ-9 scale, and anxiety scales were not evaluated comprehensively. Moreover, due to issues related to patient willingness, complete PHQ-9 data could not be obtained from all enrolled patients, potentially diminishing its predictive power for lymph node metastasis. Fourth, subjective bias may arise when patients complete questionnaires, which could further reduce predictive effectiveness. Future studies should focus on collecting comprehensive, multi-scale data to explore the predictive value of emotional distress on lymph node metastasis. Fifth, an additional limitation is the lack of long-term follow-up data. This study focused on preoperative prediction of lymph node metastasis; however, the predictive value of the nomogram for recurrence-free survival and overall survival remains unknown. Future prospective studies with extended follow-up periods are warranted to validate the long-term clinical utility of this model. Sixth, the current model does not incorporate preoperative imaging features such as TI-RADS classification or ultrasound characteristics, which have been reported to be associated with lymph node metastasis risk. This omission was intentional to prioritize the use of objective, universally accessible laboratory and clinical parameters, thereby enhancing applicability in settings where specialized thyroid ultrasound expertise is unavailable. Future iterations of the model will integrate imaging data to further optimize predictive accuracy.

## 5 Conclusions

This study analyzed data from 484 patients who sought care at the Thyroid and Neck Oncology Department at Tianjin Medical University Cancer Hospital over a three-year period. The screening process employing LASSO and multivariate logistic regression identified nine variables as independent predictors significantly associated with LNM. These variables were subsequently incorporated into a nomogram, which is expected to provide valuable clinical guidance for tailoring treatment strategies.

## Data Availability

The original contributions presented in the study are included in the article/**Supplementary Material**. Further inquiries can be directed to the corresponding authors.
