# Supplementary material for: A nomogram as a predictive tool for lymph node metastasis in papillary thyroid carcinoma
Source: Front Endocrinol (Lausanne). 2026 Jun 16;17:1850799. doi: 10.3389/fendo.2026.1850799 (PMC13314451; doi:10.3389/fendo.2026.1850799)
Supplement: Supplementary file 1 [file Table1.docx]

Supplementary Table1. The baseline characteristics of patients categorized by the presence or absence of lymph node metastasis.

| Variables | Total | Lymph-node-metastasis-No | Lymph-node-metastasis-Yes | P Value |
| --- | --- | --- | --- | --- |
|  | Total (n = 484) | 0 (n = 220) | 1 (n = 264) |  |
| **Baseline Characteristics：** |  |  |  |  |
| Age-scale, n (%) |  |  |  | < 0.001 |
| 0<x≤20 | 3 (1) | 2 (1) | 1 (0) |  |
| 20<x≤40 | 222 (46) | 76 (35) | 146 (55) |  |
| 40<x≤60 | 226 (47) | 125 (57) | 101 (38) |  |
| 60<x | 33 (7) | 17 (8) | 16 (6) |  |
| Operation-history, n (%) |  |  |  | 0.049 |
| No | 178 (37) | 70 (32) | 108 (41) |  |
| Yes | 306 (63) | 150 (68) | 156 (59) |  |
| Multiple-pregnancies-and-fertility, n (%) |  |  |  | 0.073 |
| No | 325 (67) | 138 (63) | 187 (71) |  |
| Yes | 159 (33) | 82 (37) | 77 (29) |  |
| Personal-history, n (%) |  |  |  | 0.017 |
| No | 372 (77) | 154 (70) | 218 (83) |  |
| Hypertension | 59 (12) | 37 (17) | 22 (8) |  |
| Diabetes | 14 (3) | 9 (4) | 5 (2) |  |
| Hepatitis | 6 (1) | 2 (1) | 4 (2) |  |
| Allergic history | 27 (6) | 15 (7) | 12 (5) |  |
| Hypertension and Diabetes | 6 (1) | 3 (1) | 3 (1) |  |
| Tobacco-wine, n (%) |  |  |  | 0.95 |
| No | 447 (92) | 205 (93) | 242 (92) |  |
| Smoking habit | 18 (4) | 7 (3) | 11 (4) |  |
| Drinking history | 5 (1) | 2 (1) | 3 (1) |  |
| Smoking habit and Drinking history | 14 (3) | 6 (3) | 8 (3) |  |
| Past-medical-history, n (%) |  |  |  | 0.008 |
| No | 423 (87) | 188 (85) | 235 (89) |  |
| Other thyroid disease | 15 (3) | 7 (3) | 8 (3) |  |
| Other malignancies | 13 (3) | 8 (4) | 5 (2) |  |
| Other benign tumors | 23 (5) | 16 (7) | 7 (3) |  |
| Family history of thyroid cancer | 8 (2) | 0 (0) | 8 (3) |  |
| Family history of other malignancies | 2 (0) | 1 (0) | 1 (0) |  |
| region, n (%) |  |  |  | 0.863 |
| Beijing and Tianjin and Hebei | 286 (59) | 133 (60) | 153 (58) |  |
| Dongbei | 136 (28) | 63 (29) | 73 (28) |  |
| Neimenggu | 22 (5) | 10 (5) | 12 (5) |  |
| Shandong | 19 (4) | 6 (3) | 13 (5) |  |
| Shanxi | 6 (1) | 3 (1) | 3 (1) |  |
| Shanxi | 3 (1) | 1 (0) | 2 (1) |  |
| Henan | 3 (1) | 2 (1) | 1 (0) |  |
| Hubei | 2 (0) | 0 (0) | 2 (1) |  |
| Jiangsu | 1 (0) | 0 (0) | 1 (0) |  |
| Anhui | 4 (1) | 1 (0) | 3 (1) |  |
| Gansu | 1 (0) | 1 (0) | 0 (0) |  |
| Yunnan | 1 (0) | 0 (0) | 1 (0) |  |
| Blood-type, n (%) |  |  |  | 0.619 |
| A+ | 117 (24) | 47 (21) | 70 (27) |  |
| B+ | 167 (35) | 78 (35) | 89 (34) |  |
| AB+ | 61 (13) | 28 (13) | 33 (12) |  |
| O+ | 138 (29) | 67 (30) | 71 (27) |  |
| O- | 1 (0) | 0 (0) | 1 (0) |  |
| **Pathological Characteristics：** |  |  |  |  |
| Single-Multiple, n (%) |  |  |  | < 0.001 |
| single | 282 (58) | 152 (69) | 130 (49) |  |
| multiple | 202 (42) | 68 (31) | 134 (51) |  |
| Isthmic Thyroid Cancer, n (%) |  |  |  | 0.587 |
| No | 451 (93) | 207 (94) | 244 (92) |  |
| Yes | 33 (7) | 13 (6) | 20 (8) |  |
| Bilateral Papillary Thyroid Carcinoma, n (%) |  |  |  | < 0.001 |
| No | 378 (78) | 191 (87) | 187 (71) |  |
| Yes | 106 (22) | 29 (13) | 77 (29) |  |
| Tumor with calcification, n (%) |  |  |  | 0.358 |
| No | 473 (98) | 217 (99) | 256 (97) |  |
| Yes | 11 (2) | 3 (1) | 8 (3) |  |
| Hashimoto's thyroiditis, n (%) |  |  |  | 0.199 |
| No | 380 (79) | 179 (81) | 201 (76) |  |
| Yes | 104 (21) | 41 (19) | 63 (24) |  |
| BRAF Mutation, n (%) |  |  |  | 0.425 |
| No | 205 (42) | 98 (45) | 107 (41) |  |
| Yes | 279 (58) | 122 (55) | 157 (59) |  |
| **Laboratory Characteristics：** |  |  |  |  |
| PT, Median (Q1,Q3) | 11.3 (10.9, 11.8) | 11.3 (10.8, 11.8) | 11.35 (10.9, 11.8) | 0.487 |
| TT, Median (Q1,Q3) | 16.7 (15.6, 17.3) | 16.6 (15.67, 17.3) | 16.7 (15.6, 17.4) | 0.883 |
| D-Dimer, Median (Q1,Q3) | 189.22 (124.56, 276.26) | 191.56 (126.83, 285.06) | 179.74 (120.47, 265.24) | 0.299 |
| TT3, Mean ± SD | 1.69 ± 0.27 | 1.67 ± 0.25 | 1.7 ± 0.28 | 0.395 |
| FT3, Median (Q1,Q3) | 4.92 (4.55, 5.34) | 4.89 (4.5, 5.3) | 4.97 (4.57, 5.41) | 0.22 |
| TT4, Mean ± SD | 94.97 ± 15.01 | 96.25 ± 15.74 | 93.91 ± 14.32 | 0.09 |
| FT4, Median (Q1,Q3) | 16.5 (14.88, 18) | 16.45 (14.9, 17.9) | 16.5 (14.8, 18.12) | 0.938 |
| TSH, Median (Q1,Q3) | 2.08 (1.39, 2.98) | 2.12 (1.33, 3.08) | 2.04 (1.42, 2.9) | 0.774 |
| TG, Median (Q1,Q3) | 15.3 (6.67, 28.2) | 13.4 (6.61, 25.4) | 17.65 (7.14, 32.08) | 0.021 |
| Anti-TG, Median (Q1,Q3) | 19.7 (13.4, 48.83) | 20.3 (13.2, 44.92) | 19.05 (13.6, 52.05) | 0.74 |
| Anti-TPO, Median (Q1,Q3) | 10.35 (9, 20) | 10.6 (9, 24.33) | 10.3 (9, 18.23) | 0.528 |
| PTH, Median (Q1,Q3) | 4.06 (3.25, 5.02) | 3.84 (3.16, 4.88) | 4.24 (3.34, 5.17) | 0.054 |
| CT, Median (Q1,Q3) | 0.5 (0.5, 1.08) | 0.5 (0.5, 0.9) | 0.5 (0.5, 1.27) | 0.227 |
| ALT, Median (Q1,Q3) | 15 (11, 24) | 16 (11, 23) | 15 (11, 25) | 0.777 |
| AST, Median (Q1,Q3) | 16 (13, 19) | 16 (13, 20) | 16 (13, 19) | 0.977 |
| ASTm, Median (Q1,Q3) | 6 (5, 7) | 6 (5, 7) | 6 (4, 7) | 0.455 |
| ALP, Median (Q1,Q3) | 64 (51, 77) | 65 (53, 77.25) | 63.5 (50, 76) | 0.231 |
| PALB, Median (Q1,Q3) | 0.24 (0.21, 0.27) | 0.24 (0.21, 0.27) | 0.24 (0.21, 0.27) | 0.631 |
| ALB, Mean ± SD | 43.17 ± 2.95 | 43.18 ± 2.84 | 43.17 ± 3.04 | 0.988 |
| TP, Mean ± SD | 66.67 ± 4.58 | 66.85 ± 4.28 | 66.51 ± 4.82 | 0.412 |
| GLO, Median (Q1,Q3) | 23.3 (21, 25.6) | 23.5 (21.3, 25.83) | 23.1 (20.7, 25.5) | 0.283 |
| A-G, Median (Q1,Q3) | 1.85 (1.66, 2.07) | 1.81 (1.65, 2.05) | 1.87 (1.68, 2.09) | 0.303 |
| Albumin, Median (Q1,Q3) | 60.2 (57.77, 62.5) | 59.9 (57.38, 62) | 60.4 (58.1, 62.73) | 0.089 |
| Alpha1, Median (Q1,Q3) | 3.5 (3.2, 3.9) | 3.5 (3.2, 3.9) | 3.5 (3.2, 3.9) | 0.998 |
| Alpha2, Median (Q1,Q3) | 8.6 (7.8, 9.4) | 8.65 (7.9, 9.5) | 8.5 (7.7, 9.3) | 0.219 |
| Beta1, Median (Q1,Q3) | 6.2 (5.8, 6.7) | 6.3 (5.8, 6.8) | 6.2 (5.8, 6.6) | 0.168 |
| Beta2, Median (Q1,Q3) | 4.4 (3.8, 5.1) | 4.5 (3.8, 5.12) | 4.4 (3.8, 4.93) | 0.357 |
| Gamma, Median (Q1,Q3) | 16.9 (14.9, 18.7) | 17 (15.07, 18.7) | 16.7 (14.7, 18.52) | 0.294 |
| TBIL, Median (Q1,Q3) | 9.3 (7.1, 12.4) | 9.4 (7.18, 12.3) | 9.2 (7.07, 12.43) | 0.902 |
| DBIL, Median (Q1,Q3) | 3.6 (2.9, 4.6) | 3.6 (2.9, 4.5) | 3.65 (2.9, 4.7) | 0.637 |
| GGT, Median (Q1,Q3) | 17 (12, 27) | 18 (12, 26) | 17 (12, 28) | 0.888 |
| ChE, Median (Q1,Q3) | 8197.5 (7098, 9423.5) | 8180 (7125.75, 9422.25) | 8214.5 (7065.5, 9435.25) | 0.737 |
| ADA, Median (Q1,Q3) | 6 (5, 7) | 6 (5, 7) | 6 (5, 7) | 0.735 |
| LDH, Median (Q1,Q3) | 168 (152, 184) | 166 (149, 185) | 169 (154, 183) | 0.443 |
| LD-1, Median (Q1,Q3) | 31 (29, 35) | 31 (29, 35) | 32 (29, 35) | 0.718 |
| TBA, Median (Q1,Q3) | 3.6 (2.3, 5.6) | 3.55 (2.3, 5.43) | 3.6 (2.3, 5.6) | 0.624 |
| MAO, Median (Q1,Q3) | 6.6 (4.9, 8) | 6.5 (5.1, 8) | 6.6 (4.9, 7.9) | 0.768 |
| IVC, Median (Q1,Q3) | 89.95 (83.47, 97.4) | 89.8 (84.1, 97.2) | 90.05 (82.6, 97.4) | 0.565 |
| Glu, Median (Q1,Q3) | 4.76 (4.43, 5.16) | 4.81 (4.51, 5.18) | 4.72 (4.4, 5.08) | 0.018 |
| FMN, Median (Q1,Q3) | 1.62 (1.53, 1.74) | 1.62 (1.53, 1.75) | 1.62 (1.53, 1.73) | 0.459 |
| Cr, Median (Q1,Q3) | 58 (52, 68) | 57 (51, 65) | 59 (53, 70) | 0.045 |
| UA, Median (Q1,Q3) | 290.5 (241.75, 359) | 281.5 (241, 345.5) | 303.5 (242, 363.25) | 0.084 |
| UREA, Median (Q1,Q3) | 4.35 (3.7, 5.1) | 4.35 (3.8, 5.1) | 4.35 (3.7, 5.23) | 0.557 |
| CK, Median (Q1,Q3) | 64 (50, 85) | 63 (50, 83) | 64 (50, 85.5) | 0.594 |
| CK-MB, Median (Q1,Q3) | 0.8 (0.6, 1.1) | 0.8 (0.6, 1.1) | 0.8 (0.6, 1.1) | 0.141 |
| Hcy, Median (Q1,Q3) | 9.7 (8, 11.7) | 9.4 (7.9, 11.1) | 10 (8.1, 12.12) | 0.025 |
| MB, Median (Q1,Q3) | 21 (21, 21.83) | 21 (21, 21) | 21 (21, 22.63) | 0.145 |
| TC, Median (Q1,Q3) | 4.63 (4.1, 5.27) | 4.6 (4.09, 5.26) | 4.66 (4.12, 5.27) | 0.935 |
| TG, Median (Q1,Q3) | 1.17 (0.83, 1.75) | 1.19 (0.89, 1.81) | 1.16 (0.78, 1.67) | 0.183 |
| Ca, Mean ± SD | 2.28 ± 0.09 | 2.28 ± 0.1 | 2.28 ± 0.08 | 0.785 |
| P, Median (Q1,Q3) | 1.31 (1.21, 1.42) | 1.3 (1.2, 1.4) | 1.31 (1.22, 1.43) | 0.391 |
| K, Median (Q1,Q3) | 4 (3.8, 4.2) | 4 (3.8, 4.2) | 4 (3.8, 4.2) | 0.435 |
| Na, Median (Q1,Q3) | 142 (141, 143) | 142 (141, 143) | 142 (141, 143) | 0.323 |
| CL, Median (Q1,Q3) | 106 (104, 107) | 106 (104, 107) | 106 (104, 107) | 0.575 |
| TCO2, Median (Q1,Q3) | 23 (21, 25) | 23.5 (22, 25) | 23 (21, 25) | 0.146 |
| Mg, Mean ± SD | 0.85 ± 0.07 | 0.85 ± 0.07 | 0.84 ± 0.06 | 0.884 |
| RBC, Median (Q1,Q3) | 4.46 (4.18, 4.78) | 4.46 (4.18, 4.74) | 4.46 (4.2, 4.85) | 0.317 |
| HGB, Median (Q1,Q3) | 132 (123, 143) | 132 (121.75, 140) | 133 (124, 146.25) | 0.03 |
| HCT, Median (Q1,Q3) | 40.1 (37.38, 43.02) | 39.85 (37.1, 42.3) | 40.25 (37.58, 43.62) | 0.051 |
| WBC, Median (Q1,Q3) | 5.72 (4.8, 6.66) | 5.62 (4.71, 6.64) | 5.85 (4.9, 6.67) | 0.176 |
| NEUT-R, Mean ± SD | 53.21 ± 8.1 | 52.97 ± 8.07 | 53.41 ± 8.14 | 0.56 |
| LYMPH-R, Mean ± SD | 36.5 ± 7.33 | 36.7 ± 7.29 | 36.34 ± 7.37 | 0.596 |
| MONO-R, Median (Q1,Q3) | 7 (6, 8.3) | 7.1 (6, 8.5) | 6.95 (5.9, 8.2) | 0.2 |
| EO-R, Median (Q1,Q3) | 1.9 (1.3, 3.1) | 1.8 (1.2, 3.1) | 2.1 (1.3, 3.2) | 0.197 |
| BASO-R, Median (Q1,Q3) | 0.5 (0.4, 0.7) | 0.5 (0.4, 0.7) | 0.5 (0.4, 0.7) | 0.859 |
| NEUT, Median (Q1,Q3) | 3.06 (2.43, 3.71) | 3 (2.38, 3.58) | 3.09 (2.46, 3.79) | 0.243 |
| LYMPH, Median (Q1,Q3) | 2.01 (1.68, 2.45) | 2.02 (1.66, 2.45) | 2.01 (1.71, 2.45) | 0.505 |
| MONO, Median (Q1,Q3) | 0.4 (0.33, 0.49) | 0.4 (0.33, 0.49) | 0.4 (0.33, 0.49) | 0.89 |
| EO, Median (Q1,Q3) | 0.11 (0.07, 0.18) | 0.1 (0.07, 0.17) | 0.12 (0.08, 0.19) | 0.079 |
| BASO, Median (Q1,Q3) | 0.03 (0.02, 0.04) | 0.03 (0.02, 0.04) | 0.03 (0.02, 0.04) | 0.562 |
